# Mobility-Aware Resource Allocation in IoRT Network for Post-Disaster Communications with Parameterized Reinforcement Learning

**DOI:** 10.3390/s23146448

**Published:** 2023-07-17

**Authors:** Homayun Kabir, Mau-Luen Tham, Yoong Choon Chang, Chee-Onn Chow, Yasunori Owada

**Affiliations:** 1 Department of Electrical and Electronic Engineering, Lee Kong Chian Faculty of Engineering and Science, Universiti Tunku Abdul Rahman, Sungai Long Campus, Kajang 43000, Malaysia; homayun@1utar.my (H.K.);; 2 Department of Electrical Engineering, Faculty of Engineering, Universiti Malaya, Lembah Pantai, Kuala Lumpur 50603, Malaysia; 3 Resilient ICT Research Center, Network Research Institute, National Institute of Information and Communications Technology (NICT), Tokyo 184-8795, Japan

**Keywords:** post disaster communication, internet of robotic things (IoRT), movable and deployable resource units (MDRU), deep reinforcement learning (DRL), parameterized action space, multi-pass deep Q network (MP-DQN)

## Abstract

Natural disasters, including earthquakes, floods, landslides, tsunamis, wildfires, and hurricanes, have become more common in recent years due to rapid climate change. For Post-Disaster Management (PDM), authorities deploy various types of user equipment (UE) for the search and rescue operation, for example, search and rescue robots, drones, medical robots, smartphones, etc., via the Internet of Robotic Things (IoRT) supported by cellular 4G/LTE/5G and beyond or other wireless technologies. For uninterrupted communication services, movable and deployable resource units (MDRUs) have been utilized where the base stations are damaged due to the disaster. In addition, power optimization of the networks by satisfying the quality of service (QoS) of each UE is a crucial challenge because of the electricity crisis after the disaster. In order to optimize the energy efficiency, UE throughput, and serving cell (SC) throughput by considering the stationary as well as movable UE without knowing the environmental priori knowledge in MDRUs aided two-tier heterogeneous networks (HetsNets) of IoRT, the optimization problem has been formulated based on emitting power allocation and user association combinedly in this article. This optimization problem is nonconvex and NP-hard where parameterized (discrete: user association and continuous: power allocation) action space is deployed. The new model-free hybrid action space-based algorithm called multi-pass deep Q network (MP-DQN) is developed to optimize this complex problem. Simulations results demonstrate that the proposed MP-DQN outperforms the parameterized deep Q network (P-DQN) approach, which is well known for solving parameterized action space, DQN, as well as traditional algorithms in terms of reward, average energy efficiency, UE throughput, and SC throughput for motionless as well as moveable UE.

## 1. Introduction

Due to rapid climate change, natural catastrophes, including earthquakes, floods, landslides, tsunamis, wildfires, and hurricanes, have frequently occurred worldwide, directly affecting humanity by direct and secondary deaths of human, economic, and environmental losses [1,2]. Recently, authorities have deployed various types of robots and drones such as unmanned ground vehicles (UGVs), unmanned aerial vehicles (UAVs), unmanned underwater vehicles (UUVs), mobile robots, health care robots, etc. that can be defined as user equipment (UE) for post-disaster management (PDM) because they can be dispatched to locations which cannot be accessed or too risky to be accessed by human rescuers after a disaster has occurred and yet work nonstop [3,4,5,6]. Furthermore, the Internet of Things (IoT) and robotic technologies have recently been combined in order to expand the functional capabilities of these robots, commonly called the Internet of Robotic Things (IoRT) [7,8,9,10]. The communication between IoRT devices can be provided by 4G/LTE/5G and beyond cellular communication, which can be the heterogeneous network (HetNet) [11]. Residents in the affected area are unable to express their demands and circumstances when regular IoRT/IoT networks are substantially compromised due to the disaster [12,13]. However, victims frequently require essential services, for example, food, water, medical assistance, and shelter, which must be rapidly arranged within 72 h after the disaster to save lives and mitigate losses. As a result, the immediate requirement is for rapid and effective post-disaster network rebuilding [13].

**Figure 1:**
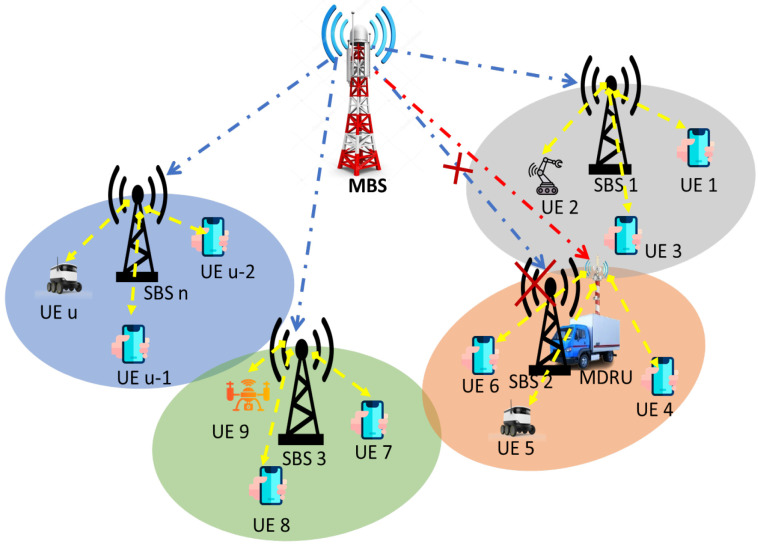
MDRU aided wireless communication after disaster.

In a post-disaster scenario, Movable and Deployable Resource Units (MDRUs) developed by Tohoku University (TU), Japan, and Nippon Telegraph and Telephone (NTT) can be adopted as SBSs to restore the network coverage and capacity due to the rapid deployment, flexibility, interoperability, and resilience [14]. On the other hand, the UAV-aided cellular network has been regarded as a crucial solution for PDM; however, UAVs can support a maximum of one hour due to the power limitation [15]. Due to the electric power unavailability for a long time in disaster-affected areas, van-type MDRU has around seven hours of battery life, similar to the battery backup of the small base station (SBS), was conducted the field test for a comprehensive solution to satisfy the demands of UEs in disaster areas [16]. In addition, the authors of [17] recommended deploying MDRUs to provide communication services where few SBSs have been interrupted due to a small disaster; in contrast, others are in working condition to reconstruct the whole cellular network. Furthermore, MDRU has been deployed to provide communication services and process the big data for minimizing the latency that is important to find injured people, animals, and damaged infrastructure in the disaster-affected area [18]. It was also used to build heterogeneous wireless IoT networks to sense, exchange, and monitor natural disasters by humanitarian organizations [19]. The authors [20] reconstructed the communication network deploying MDRU in the disaster area and found the best results in small coverage and dense area. Furthermore, the intelligent post-disaster network was developed using big crowd data. The authors deployed MDRUs connected with multiple still-alive base stations by using the virtual vertex [21]. The author in [22] recommended building a resilient IoT network by deploying MDRU, which is connected to a backbone network. In summary, all still-alive SBSs and MDRUs (replacement of damaged SBSs) are generally associated with MBS through wireless backhaul connection to handle the vast UE data generated for PDM with the quality of service (QoS). The whole system can be called two-tier HetNet, as illustrated in Figure 1.

The mobility of UEs is one of the critical points to collecting the data in the disaster-affected area for PDM, which impacts channel conditions, path loss, shadow effect, etc., and is a more realistic phenomenon. Our impression is that only a few studies have been conducted based on the mobility of UEs. In [23], we developed a twin delayed deep deterministic policy gradient (TD3) based power allocation algorithm considering UE mobility in one tier IoRT network; however, the major limitation of that research is UE association was not considered. In [24], power allocation optimization is conducted by convex optimization. However, most of the formulated problems, for example, dynamic PA, maximization of the coverage area, traffic offloading, traffic load balancing with user association, maximization of sum rate, etc., are strongly nonconvex as well as nondeterministic polynomial-time hardness (NP-hard) [25]. In this research, we investigate optimizing the energy efficiency and throughput of UE as well as serving cell (SC) of the MDRU-aided two-tier HetNet scenario by ensuring the QoS of mobility-aware UEs where user association and power allocation for each UE have been considered without knowing the environmental priori knowledge. Hence, this optimization problem is strongly nonconvex as well as NP-hard.

**Figure 2:**
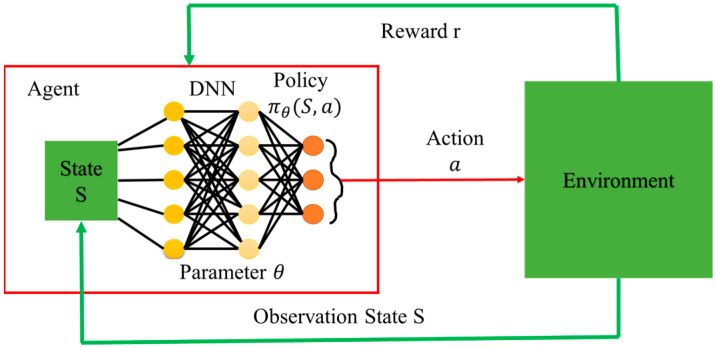
Architecture of deep reinforcement learning (DRL).

Deep Reinforcement Learning (DRL) algorithms (one of the most potent AI algorithms) can handle nonconvex and NP-hard optimization problems [25,26] by leveraging the power of deep neural networks to learn a policy that maps states to actions. The reinforcement learning framework provides a way to learn this policy by trial and error through interaction with the environment illustrated in Figure 2. By learning from experience, the agent can gradually improve its performance and find suitable solutions to complex optimization problems. Consequently, DRL has been applied in wireless communication, robots, computer vision, IoT, IoRT, etc. [27]. According to the action space, DRL is classified as discrete action space algorithms, for example, Deep Q network (DQN), Double DQN (DDQN), Rainbow DQN, dueling DQN, etc., continuous action space algorithms; for instance, Deep Deterministic Policy Gradient (DDPG), Twin delayed DDPG (TD3), Distributed Distributional DDPG (D4PG), Soft Actor-Critic (SAC), etc., that are based on policy gradient and hybrid action space algorithms, such as Q-PAMDP, PA-DDPG, Parametrized DQN (P-DQN), Multi-Pass DQN (MP-DQN), etc., that can handle discrete-continuous combinedly [28].

In [29], authors investigated a combined strategy for power allocation, which is considered continuous action and user association that is discrete action to improve downlink energy efficiency while ensuring QoS of stationary UEs under standard backhaul connection in HetNet by implementing the hybrid action space-based DRL called P-DQN. Furthermore, the architecture of P-DQN is similar to DDPG. However, discrete action is produced from the Q network, while continuous action is generated from the actor network. In P-DQN, the joint action parameter vector for all whole actions at a one-time step is taken as the input of the Q network. As a result, the gradients concerning all action parameters are calculated, which generates false gradients. In [30], the authors developed MP-DQN (similar architecture as P-DQN) and tested it in well-known Robot Soccer Goal and Half Field Offense games. They forwarded the continuous action parameter with a standard basis vector to the Q-network. Consequently, it mitigated the effects of false gradients of P-DQN, converged the complex optimization problem, and outperformed P-DQN regarding data efficiency and converged policy performance [28,30]. After that, researchers are applying MP-DQN to solve hybrid action space-based optimization problems in various fields from robotics to communications. MP-DQN was implemented in the Golf simulator to find the best action that consisted of shot selection with height, spin, and speed [31]. In [32], the authors implemented MP-DQN to build a decision tree method for the imbalanced binary classification where the continuous attributes represented the discrete action, and the threshold values were continuous action. For intelligent traffic signal control development, MP-DQN was deployed in [33], considering selecting traffic lights (red, amber, and green) with on–off time intervals. In [34], the authors applied the MP-DQN in an actual robot setup to primitive action (translation, rotation, and insertion) with the end-effector velocity as well as the contact force limits. In wireless communication, MP-DQN has been implemented for task scheduling of the Radio Access Network [35] and joint task offloading and resource allocation in the Non-orthogonal multiple access (NoMA) system [36].

This paper explores MDRU aided two-tier HetNet scenario considering the UE mobility for post-disaster communication. It aims to optimize resource allocation by deploying the parameterized DRL called MP-DQN without knowing the environmental priori knowledge. The main contributions of this article are summarized below:

1. We investigate UE association and power allocation for maximizing the energy efficiency of downlink in MDRUs-aided two-tier HetNet for post-disaster communications by considering the backhaul links of MDRUs/SBSs with MSB where UE association as discrete action space and power allocation as continuous action space combinedly called parameterized action space of DRL has been considered when UEs are stationary.
2. Mobility-aware resource allocation (UE association and power allocation) has been formulated for parameterized DRL to optimize the energy efficiency, the throughput of SBSs/MDRUs, and the throughput of UEs in MDRUs aided two ties HetNet.
3. Model-free and parameterized action space-based MP-DQN algorithm, which utilizes several concurrent batch processing to provide action parameters to the Q network, has been proposed to maximize the energy efficiency, the throughput of SBSs/MDRUs and throughput of UEs of MDRUs aided HetNet.

Note that the proposed framework improves network robustness, which is one of the goals of the ASEAN IVO project titled “Context-Aware Disaster Mitigation using Mobile Edge Computing and Wireless Mesh Network”.

## 2. Related Work

TU, Japan, and NTT are conducting research continuously to improve the MDRU performance in terms of connectivity, serviceability, and coordination during PDM. They deployed the channel allocation algorithm in MDRU and conducted the test successfully in the Philippines and Nepal [37]. Due to the limited power after the disaster, emitting power optimization of rapidly deployable resource units by satisfying the demand of UEs (search and rescue robots, drones, smartphones, etc.) has paid great attention [38]. In [39], authors proposed radio access control based on DRL for selecting the van type MDRUs/relay and optimizing the power of MDRUs. In [40], authors investigated spectrum and energy-efficient methods for providing communication services to UE of MDRU-based networks. The authors [41] analyzed the problem of UAV deployment as MDRU in both standalone deployment scenarios to support fixed SBSs already in place where SBSs are damaged due to malfunction or disaster in HetNeT. In addition, they considered that UAVs were connected with remaining SBS or MBS by wireless backhauls, which was essential to serve the UEs by fulfilling their demands.

In [24], we implemented DRL, consisting of two value-based networks for energy-efficient radio resource allocation in IoRT that outperformed the DQN [42], where the UE demand and status (active or sleep) of BS are considered as state and action is to estimate the status of each BS. In addition, emitting power of active BS to serve UEs was optimized by a convex optimizer. In [43], value-based distributed DRL has been proposed to find user association and resource allocation by ensuring UE QoS. After that, the simulation results were improved by implementing the D3QN consisting of DDQN and dueling architecture in [44], where the degree of satisfaction of UEs was state space, and the selection of BS and transmission channels combinedly were action space. However, emitting power of BS can be adequately optimized when emitting power is conserved as continuous action of the DRL algorithm. In [23], continuous action-based DRL algorithms, TD3, have been applied to estimate the optimal emitting power of BS in the IoRT network by considering the interfering multiple access channel (IMAC). In [45], the authors developed a novel DRL based on DDPG to optimize the joint issue user association and power allocation of BS in HetNet that achieved the load balance and improved the energy efficiency of the network. In [46], a transfer learning algorithm based on DDPG has been developed to optimize the power allocation and ensure user association in HetNet. However, user association is discrete and power allocation is continuous. Hence, to solve the joint optimization problem combined with user association and power allocation, a hybrid action space-based DRL algorithm is the most suitable. In [29], the author formulated the problem of combined user association and power allocation, where user association considers as the discrete action and power allocation is expressed as the continuous action. In addition, P-DQN has been implemented to maximize energy efficiency by satisfying the QoS of each UE. Simulation results of P-DQN outperformed compared to DQN in terms of overall efficiency by satisfying the QoS of stationary UEs.

For PDM, UEs need to move in the vicinity to collect the appropriate information about victims that movement directly affects communication channel quality and throughput. This critical phenomenon has not been taken into account by many academics. The UE mobility model in non-orthogonal multiple access (NOMA), where each UE moved from one point to another with varied directions and speeds, was taken into consideration by the authors [47]. Due to UE mobility, the authors [48] suggested a conventional dynamic power allocation (DPA) method that took the channel circumstances into account and asserted that UE mobility significantly influences NOMA’s performance, particularly for downlink throughput. The authors of [49] created a power control method for a wireless network where UE mobility causes changes in the communication channel. In [23], two widespread UE mobility models, (a) modified Gauss–Markov and (b) random walk, have been investigated to maximize the sum rate in dynamic power allocation problems where the TD3-based DRL algorithm has been implemented; however, user association was not considered. In [50], the authors implemented Genetic Algorithm (GA) to allocate the UEs worked to share the information after the disaster in overlapping areas among the appropriate MDRUs. The proposed GA algorithm outperformed greedy and random algorithms as well as the nearest MDRU in terms of latency and QoS. In order to maximize the expected achievable rate of UE in ultra-dense networks, the authors [51] developed a matching game algorithm, where mobility-aware user association was considered by minimizing the handovers number. The authors [52] deployed the DRL algorithm to estimate the transmit timing, routing as well as power allocation for UEs from MDRU deployed in disaster areas where UE mobility, channel states, and energy harvesting were considered.

## 3. System Model

In this section, we consider two-tier HetNet that consists of one MBS with M active SBSs and N deployed MDRUs (replacement of damaged SBSs due to disaster) where M={1,2,…,M} and N={1,2,…,N} are the sets of active SBSs and deployed MDRUs [17,20,50]. The total SCs for PDM is K=M+N where K={1,2,…,K} is the set of SCs that serve U UEs considering U={1,2,…,U} is the set of UEs. In addition, we assume two different bands that are 6 GHz and millimeter wave bands for MBS to SBSs/MDRUs (tier 1) and SCs to UEs (tier 2), respectively. As a result, interference between tiers is not available in this network. For tier 1 downlinks, the antenna array of MBS is larger than the total number of SCs. Furthermore, orthogonal frequency division multiple access (OFDMA) is deployed to communicate from SBSs/MDRUs to UEs where the total subchannels number is $N_{sub}$. To collect the data and survey the disaster-affected area, UEs need to move from one place to another. Hence, the mobility model of UE has to be considered for PDM. Modified Gauss–Markov is the well-known mobility model of UE, especially for robots and drones that are considered in our research.

### 3.1. Modified Gauss-Markov Mobility Model

**Figure 3:**
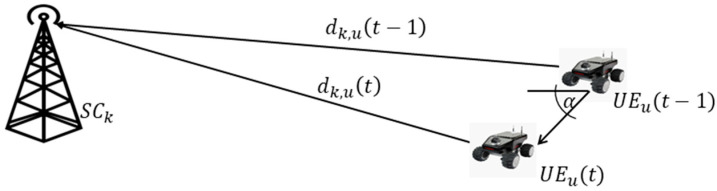
UE Mobility model for UE with random direction and average velocity.

The Modified Gauss–Markov (MGM) mobility model improves past approaches by including temporal dependence. Here, the speed and direction of a UE are updated in line with the recorded values of earlier time periods. The degree of randomness used in calculating these two figures can also be changed based on the features of the simulated wireless network. The MGM mobility paradigm is not stateless since the memory of past actions are retained. Nonetheless, the UE mobility continues to be distinct from that of other mobile terminals linked to the same network [47,53]. According to Figure 3, UE mobility makes possible $u^{th}$ UE to move randomly with average velocity that is indicated as ${\Delta \alpha }_{u}(t-1,t)$ and $v_{u}(t-1,t)$, $u^{th}$ UE are $x_{u}(t)$ $y_{u}(t)t$ are presented below:

(1) $$x_{u}(t)=x_{u}(t-1)+v_{u}(t-1,t)*\cos\left(\alpha_{u}(t-1,t)\right)*\Delta t,$$

(2) $$y_{u}(t)=y_{u}(t-1)+v_{u}(t-1,t)*\mathrm{sing}\left(\alpha_{u}(t-1,t)\right)*\Delta t,$$

(3) $$\alpha_{u}(t)=\alpha_{u}(t-1)+{\Delta \alpha }_{u}(t-1,t),$$

where $x_{u}(t-1)$, $y_{u}(t-1),$ and $\alpha_{u}(t-1,t)$ are the *x*-axis, *y*-axis, and direction of $u^{th}$ UE at t−1 time slot. The distance traveled by $u^{th}$ within Δt can be illustrated by

(4) $$d_{u}(t-1,t)=\sqrt{{\left(x_{u}(t-1)-x_{u}(t)\right)}^{2}+{\left(y_{u}(t-1)-y_{u}(t)\right)}^{2}}.$$

The distance between $k^{th}$ SC and $u^{th}$ UE at t time slot is presented as

(5) $$d_{k,u}(t)=\sqrt{{\left(x_{k}-x_{u}(t)\right)}^{2}+{\left(y_{k}-y_{u}(t)\right)}^{2}},$$

where $x_{k}$ and $y_{k}$ are the coordinates of $k^{th}$ SC.

### 3.2. Network Model

Even though each SBS/MDRU are using OFDMA to serve UEs that construct a cluster of UEs, each UE can only be connected to a single SC. Let’s consider $k^{th}$ serving cell serves to $u^{th}$ UE by $F_{r}$ frequency subchannel. Here, $c_{ku}(t)=\left\{0,1\right\}$ is represented as the status of user association where $c_{ku}(t)=1$ denotes the $u^{th}$ UE is associated with $k^{th}$ SC and $c_{ku}(t)=0$ otherwise. After that, the set of UEs in the cluster k is assumed by $C_{k}(t)=\{u:c_{ku}(t)=1,\;u\epsilon U\}$. The SBS serves the $u^{th}$ UE can be illustrated by $S_{u}(t)=\{k:c_{ku}(t)=1,\;m\epsilon M\}$ where ${|S}_{u}(t)|$ is one. The set of active SC is at t time slot $K^{active}(t)=\{k||C_{k}(t)|>0\}$. The channel gain between from $k^{th}$ SC to $u^{th}$ UE can be defined as

(6) $$g_{k,u,f}(t)={\left|h_{k,u,f}(t)\right|}^{2},$$

where $h_{k,u,f}(t)$ is the channel coefficient when subchannel frequency is f. The signal to interference plus noise ratio (SINR) from $k^{th}$ serving cell to $u^{th}$ UE can be illustrated as follows:

(7) $$SINR_{uf}(t)=\frac{\sum_{k=1}^{K}c_{k,u}(t)g_{k,u,f}(t)p_{k,u,f}(t)}{\sigma^{2}+I_{u,f}(t)},$$

where $p_{k,u,f}(t)$ is the allocated power of $k^{th}$ SC for $u^{th}$ UE which must be satisfied the $0\leq \sum_{u\in C_{k}}\sum_{f\in F_{R}}p_{k,u,f}(t)\leq P_{{SC}_{k},max}$. $P_{{SC}_{k},max}$ is the maximum emitting power from $k^{th}$ SC. The observed interference and noise power by $u^{th}$ UE is $I_{u,f}(t)$ and $\sigma^{2}$ respectively. To ensure no intra-cluster interference in each cluster, we investigate the case in which each SC allots orthogonal subchannels to various UEs within its serving area. Every UE can receive a minimum of one subchannel to transmit the data for data transmission when the cluster size does not exceed the total number of sub-channels. When intra-cluster interference is absent, just inter-cluster interference makes up the interference term $I_{u,f}$ which may be represented as

(8) $$I_{u,f}(t)=\sum_{w\notin C_{S_{u}}}\sum_{f\in F_{u}\cap F_{w}}g_{k,u,f}(t)p_{k,w,f}(t).$$

The spectral efficiency of the $u^{th}$ UE is illustrated as

(9) $$\rho_{u}(t)=\sum_{f\in F_{k}}\log_{2}\left(1+SINR_{u,f}(t)\right).$$

The user sum-rate for the $k^{th}$ SC is calculated as

(10) $$\rho_{k}^{SC}(t)=\sum_{u\in C_{k}}\rho_{u}(t)=\sum_{u=1}^{U}c_{k,u}(t)\rho_{u}(t).$$

The summation of data transmission power and the operating power that is defined as the minimum power requirement to maintain the SC active is defined total consumed power of our network that can be represented as

(11) $$P_{total}(t)={|K}^{active}(t)|.P_{o,SC}+\sum_{k\in K}\sum_{u\in C_{k}}\sum_{f\in F_{k}}p_{k,u,f}(t).$$

where $P_{o,SC}$ is the operational power of SC. Detailed notation descriptions are summarized in Table 1.

We strive for a way that results in optimizing user association and emitting power allocation to maximize the energy efficiency expressed as the achievable sum rate per utilized power in our assumed network by considering the QoS guarantee, and wireless backhaul link capacity constraints without knowing the environmental priori knowledge. The problem can be formulated as

(12a) $$\overset{max}{c_{ku}(t),p_{k,u,f}(t)}\sum_{t=0}^{t=T}\frac{1}{P_{total}(t)}\sum_{u=1}^{U}\rho_{u}(t),$$

(12b) $$\mathrm{Subject}\;\mathrm{to}\;C_{1}:\sum_{k}c_{k,u}(t)=1,c_{k,u}(t)\in \left\{0,1\right\},\forall_{k}\in K,u\epsilon U,$$

(12c) $$C_{2}:0\leq \sum_{u\in C_{k}}\sum_{f\in F_{R}}p_{k,u,f}(t)\leq P_{{SC}_{k},max},\forall k\in K,u\epsilon U,$$

(12d) $$C_{3}:\rho_{u}(t)\geq v_{u},\forall u\epsilon U,$$

(12e) $$C_{4}:\left|C_{k}(t)\right|\leq {\left|C_{k}\right|}_{max},\forall_{k}\in M,$$

(12f) $$C_{5}:\rho_{k}^{SC}(t)\leq D_{k}^{SC},\forall_{m}\in M.$$

Each UE is presumed to be serviced by a single SC in $C_{1}$ in (12b), and the transmit power limit at the $k^{th}$ SC is discussed in $C_{2}$ in (12c), where $P_{{SC}_{k},max}$ is the maximum power that is used at the $k^{th}$ SC. $C_{3}$ in (12d), where $\upsilon_{u}$ is the capacity threshold for $u^{th}$ UE denotes the QoS requirement for each UE. The cluster size limitation in (12e) is $C_{4}$, and the maximum number of users in k cluster is ${|C}_{k}|max$. To prevent intra-cluster interference, this makes sure that UEs in the same cluster are given distinct subchannels. $D_{k}^{SC}$ is the highest feasible downlink data rate for $k^{th}$ SC, while $C_{5}$ in (12f) is the backhaul connection capacity restriction.

By identifying the best user associations as well as power distribution, which is often a difficult task with a variety of unknowns and hybrid unknown spaces (discrete clustering and continuous power) in the network, the technique in (12a) aims to maximize energy efficiency. Additionally, the optimization issue in (12a) involves a one-shot situation at a certain time instant that must be reassessed as the network advances until the following time instant. We are consequently driven to deploy MP-DQN approaches to address the issues.

## 4. Deep Reinforcement Learning for Parameterized Action Space

In this section, we illustrate the DRL which can handle the parameterized action space for identifying optimal user association (discrete action) as well as emitting power allocation (continuous action) of SC by satisfying the QoS. The parameterized action space [54] combined with discrete and continuous action space represented as $A_{d}$ and $A_{j}$ respectively is illustrated as $A=\{(j,z_{j})|z_{j}{\in A}_{j}\;for\;all\;j\in A_{d}\}$, where $a(t)=(j,z_{j})$ is the hybrid action. A discrete action j has been chosen from the discrete action set $A_{d}=\left\{j_{1},j_{2},j_{3},\ldots ,j_{J}\right\}=\{\left[c_{ku}(t)\right]:c_{ku}(t)=\left\{0,1\right\},k\in K,u\epsilon U\}$. The continuous action parameters for that specific discrete action j is $z_{j}=p^{UE}(t)=[p_{1}^{UE}(t),p_{2}^{UE}(t),\ldots ,p_{u}^{UE}(t)]$, where $p_{u}^{UE}(t)={\left[p_{S_{u},u,f}(t)\right]}_{f:f\in F_{u}}$ for downlink data transmission in all sub channels allocated to $u^{th}$ UE. Furthermore, $z_{j}\in Z$, where Z is the set of continuous actions considering all possible discrete action. According to [55], parameterized action MDP (PAMDP) is presented as <S,P,A,R,γ>. Here, S represents the state space, the Markov probability of transition is illustrated as P, the parameterized action space is denoted by A, the reward is defined R and the discount faction is γ∈[0,1]. At the $t^{th}$ timeslot, the agent observes the state of environment s(t)∈S and chooses suitable parameterized action a(t)∈A based on its policy π. After applying the chosen parameterized action, the immediate reward r(s(t),a(t)) is received with next state of environment s(t+1)~P(s(t+1)|s(t),a(t)).

To solve the non-convex, the NP-hard and joint optimization problem consists of selecting the user association and allocating the transmitted power of MDRU-aided two-tier HetNet discussed in Section 3 by parametrized DRL, state, action, reward, and experience replay are described below:

State: The data rate of each UE at $t^{th}$ timeslot has been generated from SINR that is calculated considering the user association, emitting power allocation, channel gain, interference, and noise poser observed by UE in that specific time slot. Hence, the set of data rate for all UE has been assumed as the state at $t^{th}$ timeslot for DRL agent.

(13) $$s(t)=\left[\rho_{1}(t),\ldots ,\rho_{u}(t)\right].$$

Action: In this optimization problem, discreate (identification of UE association) and continuous (emitting power for each UE from SBS) action spaces at $t^{th}$ timeslot have been combinedly considered as follows:

(14) $$a(t)=\left[c^{UE}(t),p^{UE}(t)\right],$$

where $c^{UE}(t)=\left[c_{ku}(t)\right],k=1:M,u=1:U$ with $c_{ku}(t)=\left\{0,1\right\},k\in K,u\epsilon U$ is denoted for UE association with SC. When $c_{ku}(t)=1$, it means $u^{th}$ UE is associated with $k^{th}$ SC for that specific time slot and otherwise $c_{ku}(t)=0$. After ensuring the UE association, SC is allocation power to that UE at $t^{th}$ timeslot. The vector of power allocation from SCs at $t^{th}$ timeslot is defined as $p^{UE}(t)=[p_{1}^{UE}(t),p_{2}^{UE}(t),\ldots ,p_{u}^{UE}(t)]$.

Reward: The maximization of the overall energy efficiency according to the Equation (12a) is the prime goal of this research by satisfying the QoS of every UE and the constraint capacity of backhaul link of each SBS. Therefore, the reward r(t) at $t^{th}$ time slot is illustrated as:

(a) Reward function one (RFO) [29]:

(15) $$r(s(t),a(t))=\left\{\begin{array}{c} r^{\prime }(s(t),a(t))\;\;if\;\rho_{k}^{SC}\leq R_{k}^{SC},\forall_{k}\in K\\ r^{\prime }(s(t),a(t))-r_{th}\;\;\;if\;\rho_{k}^{SC}>R_{k}^{SC}\;for\;some\;k\in K, \end{array}\right.$$

where $r^{\prime }(s(t),a(t))≅\lambda_{1}Z_{\alpha_{1}(t)}-\lambda_{2}Z_{\alpha_{2}(t)}$ with $\alpha_{1}(t)=\frac{1}{P_{T}}\sum_{u=1}^{U}\rho_{u}(t)$ that is the energy efficiency of system and $\alpha_{2}(t)=\sum_{u=1}^{U}{\left(\rho_{u}(t)-\nu_{u}\right)}^{2}$ which is the penalty term is deployed for discouraging the agent to take the actions, for example, the capacity of every UE huge diverges from the threshold of QoS and $Z_{\alpha_{1}(t)}$ and $Z_{\alpha_{2}(t)}$ are the Z-scores of $\alpha_{1}(t)$ and $\alpha_{2}(t),$ respectively. In addition, $r_{th}$ is the threshold value is deployed to mitigate the likelihood of violating the backhaul capacity constraint.

(b) Reward function two (RFT):

(16) $$r(s_{t},a_{t})=\lambda_{1}Z_{\alpha_{1}(t)}+\lambda_{2}\sum_{u=1}^{U}R_{QoS\_UE}(t)+\lambda_{3}\sum_{m=1}^{M}R_{QOS\_backhaullink}(t),$$

$$\alpha_{1}(t)=\frac{1}{P_{T}}\sum_{u=1}^{U}\rho_{u}(t),\;R_{QOS_{UE}}(t)=if\;\rho_{u}\geq v_{u},r=1\;else\;r=-1,\;R_{QOS_{backhaullink}}(t)=if\;\rho_{k}^{SC}\leq D_{k}^{SC},r=1\;else\;r=-1.$$

Here, $\lambda_{1}$, $\lambda_{2}$, $\lambda_{3}$ are non-negative weights of the corresponding terms and range from 0 to 1.

Experience replay: It is a DRL strategy that utilizes replay memory to record the agent’s experiences at each time step in a data set that is pooled over several episodes. After that, a minibatch of experience is selected randomly from the experience replay that is utilized for training. This process solves the problem of autocorrelation leading to unstable training.

Furthermore, three well-known DRL including our proposed method called MP-DQN which can handle parameterized action space are discussed below.

### 4.1. Deep Q Netwrok

One of the most well-known DRL algorithms is DQN [56], which is value-based and utilized for discrete action space only. The goal of traditional DQN is to find optimized the action by maximizing the action value function $Q_{\left(s,a\right)}$ as follows:

(17) $$Q(s,a)≙E\left[\sum_{k=0}^{\infty }\gamma^{k}r(s(t+k),a(t+k))|s(t)=s,a(t)=a\right].$$

The maximization of (17) is equivalent to the Bellman equation and can be described as

(18) $$y(t)=r(t)+\gamma {max}_{a(t+1)}Q(s(t+1),a(t+1);\theta^{-}),$$

where y(t) represents the optimized value of Q. The loss function is represented as

(19) $$L={\left(y(t)-Q(s(t),a(t);\theta )\right)}^{2},$$

which mitigates the correlation between current value Q(s(t),a(t);θ) and target value y(t). In addition, the traditional DQN can be deployed for continuous action space when it is converted into a finite set of discrete action spaces by discretizing the process. Furthermore, DQN can also be utilized for parameterized action space by converting from continuous to discrete action space that concatenates with existing discrete action space. When continuous action has conducted the quantization to reverse discrete action, many action values are generated and those action values may round off. Consequently, the complexity of the DQN exponentially rises with the size of the action space, resulting in very massive power consumption and a delay in convergence speed. To overcome those issues, P-DQN has been deployed to handle the parameterized action space-based optimization problem [28].

### 4.2. Paramataized Deep Q Learing

P-DQN [57] is a DRL algorithm that handles hybrid (discrete-continuous) action spaces combined without relaxation or approximation. The structure of P-DQN is similar to DDPG, which describes a deterministic function that takes the state as input and produces continuous parameters of each discrete action. After that, generated continuous action parameters are concatenated with the state, which is utilized as input to the Q network to generate the Q values. Finally, the optimal function chooses the best discrete action from generated Q values. Let’s consider one actor parameter network $z_{j}(s;\theta )$ with weight θ and one actor network $Q(s,j,z_{j};w)$ with weight w. Furthermore, the weights θ has been estimated by optimizing the expected function of the action-value that are described as $E[Q(s,j,z_{j}(j;\theta );w)]$. And the weight w has been determined by optimizing the mean squared error $E[{\left(y(t)-[Q(s(t),a(t);w)]\right)}^{2}]$, where $a(t)=(j,z_{j})$ and the target value is described as

(20) $$y(t)=r(t)+\gamma \;\underset{j^{\prime }\in A_{d}}{\max}Q(s(t+1),j^{\prime },z_{j}^{\prime }(s(t+1);\theta^{-});\omega^{-}).$$

In addition, the loss function of the actor parameter and the actor network can be presented as follows:

(21) $$L^{x}(\theta )=\frac{1}{N}\sum_{t=1}^{N}Q(s(t),j,z_{j}(s(t);\theta );\omega ),$$

(22) $$L^{Q}(\omega )=\frac{1}{N}{\sum_{t=1}^{N}\left(y(t)-Q(s(t),j,z_{j}(s(t);\theta );\omega \right))}^{2}.$$

Furthermore, the weights *θ* and *ω* are updated according to

(23) $$\theta \leftarrow \theta -\alpha_{a,p}{𝛻}_{\theta }L^{x}(\theta ),$$

(24) $$\omega \leftarrow \omega -\alpha_{a}{𝛻}_{\omega }L^{x}(\omega ),$$

where $\alpha_{a,p}$ and $\alpha_{a}$ are the learning rate for the actor parameter and actor network.

Even if P-DQN can converge and the impact is excellent, there is still room for improvement in the theory behind discrete and continuous action selection. Updates to any action’s continuous action parameter will affect all actions’ Q values, not just the Q value linked to the action parameter [28,30].

### 4.3. Multi Pass Deep Q Learing

The issue of excessive parameterization of P-DQN is resolved by MP-DQN [30] by employing multiple concurrent batch processing to provide action parameters to the Q network. Without altering the P-DQN structure, MP-DQN isolates the continuous parameters and inputs each one into the Q network individually. They executed a forward pass once for each discreate action j where the state s and action parameter vector $ze_{j}$ are concatenated as input and $E_{j}$ represents the j dimensional standard basis vector. Hence, the joint parameter vector is represented as $ZE_{j}=(0,\ldots ,0,z_{j},0,\ldots ,0)$ where each $z_{i},i\neq j$ is set to zero. As a consequence, the impact of network weights is negated for unassociated action parameters $z_{j}$ from the input layer where all false gradients are set to zero. Furthermore, Q is only depended on associated $z_{j}$ where

(25) $$Q(s,j,ZE_{j})≊Q(s,j,z_{j}).$$

**Figure 4:**
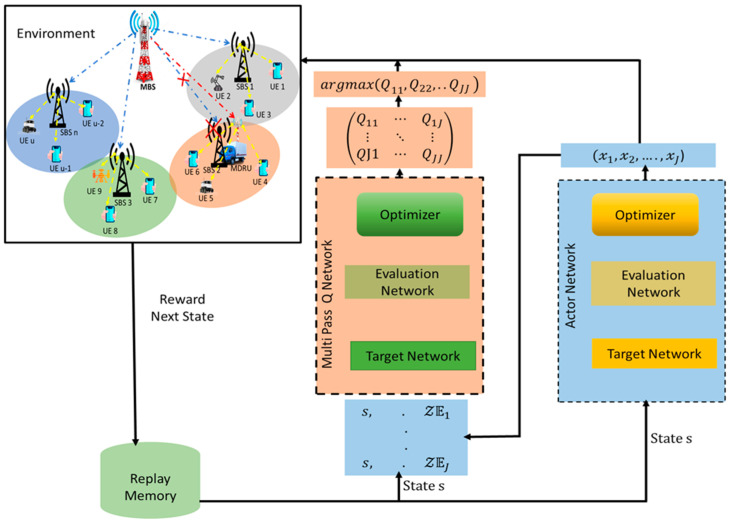
Architecture of multi pass deep Q learning (MP-DQN).

To forecast all Q values, c forward passes are necessary as opposed to just one. To perform the multi pass, the capacity of parallel minibatch processing by PyTorch or Tensorflow library can be deployed. A multi-pass with j actions is processed in the same manner as a minibatch of size j:

(26) $$(\begin{array}{c} Q(s,.,ZE_{1};\theta_{Q})\\ .\\ .\\ .\\ Q(s,.,ZE_{J};\theta_{Q}) \end{array})=(\begin{array}{ccc} Q_{11} & \cdots & Q_{1j}\\ \vdots & \ddots & \vdots \\ Qj1 & \cdots & Q_{JJ} \end{array}),$$

where the Q-value for action b produced on the $a^{th}$ pass is $Q_{ab}$. Furthermore, the diagonal elements $Q_{aa}$ is pivotal and deployed in the final output ${Q_{a}\leftarrow Q}_{aa}$ as shown in Figure 4. According to [33], MP-DQN makes it easier to choose the best hybrid action by reducing the impact of a single discrete action on other continuous action parameters.

## 5. Performance Evaluations

**Figure 5:**
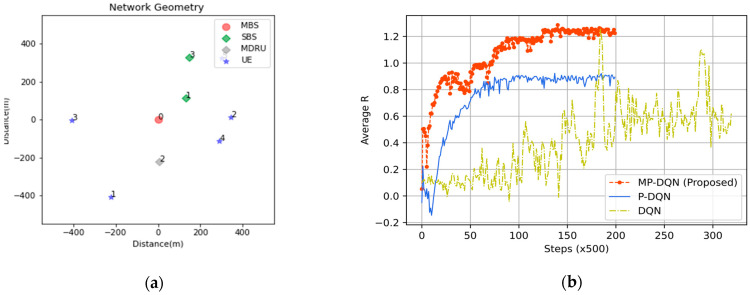
(**a**) Network geometry of HetNet based IoRT network and (**b**) Average reward (normalized) of proposed method (MP-DQN), P-DQN and DQN during the training session.

In this section, we utilize TensorFlow 1.14.0 on Spyder IDE 3.3.6 in an 11th Gen inter-core i7, 16 GB RAM, and RTX 3060 laptop GPU to demonstrate the simulation scenario. In addition, a HetNet has been considered which consists of one MBS with 100 antennas and 20 beamforming groups, three SBSs connected with MBS through backhaul transmission model [58], and five UEs presented in Figure 5a. We consider the non-line-of-sight path-loss model for urban MBSs and SBSs [59] and slow Rayleigh fading channels h~CN(0,1). We followed the same system configuration as [29] for ensuring a fair comparison, tabulated in Table 2. All simulation results have been standardized by using the Z-score.

We compare the proposed MP-DQN presented in Algorithm 1 for the optimization problem, where UE association and emitting power allocation of SBSs/MDRUs have been considered jointly as the hybrid action space with two DRL based algorithms P-DQN and DQN. For DQN, continuous action space is converted into discrete by quantization process with $\frac{P_{{SC}_{m},max}}{10^{L}}$, where L is the discrete power levels (L=5 is considered in our simulation). In addition, the simulation results of the proposed method are compared to a well-known method called Nearest SBS/MDRU with Random Power. Each UE is connected to the nearby SC, which generates random power to serve every UE in its SC’s cluster by fulfilling the conditions (1) the total power for all UE must be less or equal to the maximum power and (2) the total sum rate cannot exceed backhaul capacity of each SBS/MDRU. Furthermore, we consider the size of replay memory is 20,000, mini batch is 128, and the discount factor is 0.95 for all DRL algorithms. The total episodes for MP-DQN and P-DQN are 2000 while each episode has 50 timesteps. However, 3200 episodes are considered to simulate DQN. It takes more episodes to converge in hybrid action space-based optimization problem. In addition, other hyperparameters for MP-DQN, P-DQN, and DQN are tabulated in Table 3.
**Algorithm 1:** Multi pass DQN (MP-DQN) Algorithm.**Input**: Probability distribution ξ, mini batch size B, exploration parameter ε, learning rates $\left\{\alpha_{a},\alpha_{a,p}\right\}$. **Initialization**: actor weights $\omega ,\omega^{-}$ and actor parameter weights ($\theta ,\theta^{-}$) **For**
*t* = 1, 2, 3, *T* **do**   Estimate the action parameters $z_{j}(s(t);\theta (t))$ by actor network   Choose the action $a(t)=(j,z_{j})$ based on the ε greedy policy:      $a(t)=\left\{\begin{array}{l} radom\;sample\;according\;to\;probability\;distribution\;\xi ,\;with\;\varepsilon \\ \left(j,z_{j}):j={argmax}_{c\in A_{d}}Q(s(t),j,ze_{j};\omega ),\;\;\;with\;(1-\varepsilon \right) \end{array}\right.$   Execute action a(t), receive immediate reward r(s(t),a(t)) and next state s(t+1)   Save the experience (s(t),a(t),r(t),s(t+1)) into replay memory   Select mini batch size B randomly from the replay memory   Define the target y(t) by       $y(t)=r(t)+\gamma \underset{j^{\prime }\in A_{d}}{\max}Q(s(t+1),j^{\prime },z_{j}^{\prime }(s(t+1);\theta^{-});\omega^{-})$   Select the diagonal element from $\left(\begin{array}{ccc} Q_{11} & \cdots & Q_{1c}\\ \vdots & \ddots & \vdots \\ Qc1 & \cdots & Q_{cc} \end{array}\right)$   Choose the best action j by argmax from diagonal elements   Use the (y(t),s(t),a(t)) to estimate the gradients ${𝛻}_{\omega }L^{x}(\omega )$ and ${𝛻}_{\theta }L^{x}(\theta )$ Update the weights parameters $\omega ,\omega^{-},\theta ,\theta^{-}$


### 5.1. Simulation Results for Stationary UEs

We illustrate the average normalized results versus step over 500 realizations of proposed MP-DQN, P-DQN, and DQN algorithms during the training session. Figure 5b presents the average normalized reward of proposed MP-DQN, P-DQN and traditional DQN. Due to the complexity of the traditional DQN for discretization issues and the size of action space, the average reward is not properly converged. In P-DQN, the results are perfectly converged and saturated after-time steps. The final value of the average normalized reward is around 0.91. In comparison, the results of the proposed MP-DQN have converged perfectly but are a saturated bit later than P-DQN. The saturated value of the proposed method is around 1.25, which is clearly best compared with P-DQN and DQN algorithms.

**Figure 6:**
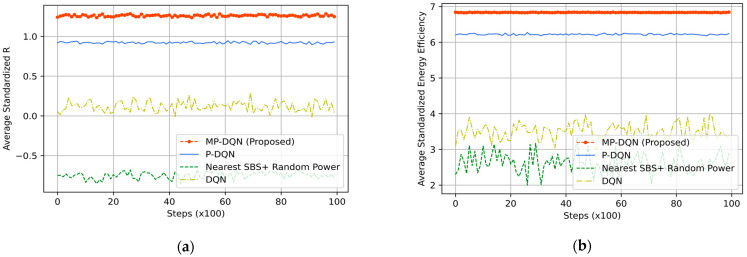
(**a**) Average reward (normalized) and (**b**) Average energy efficiency (normalized) of proposed method (MP-DQN), P-DQN, and DQN during the test session.

**Figure 7:**
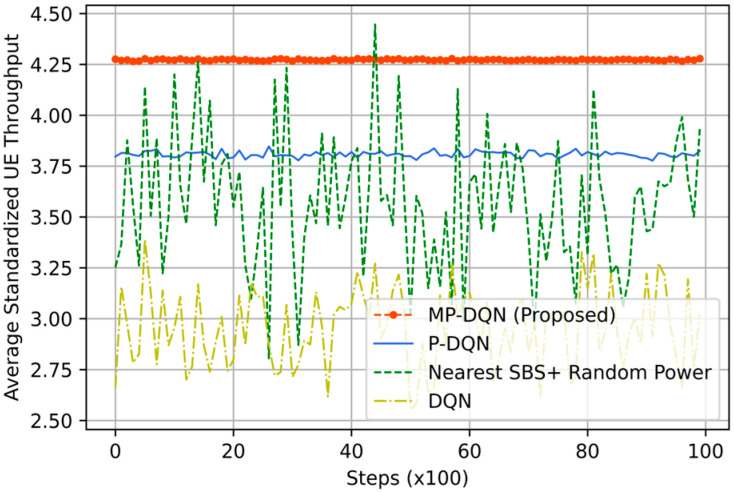
The average normalized system throughput proposed method (MP-DQN), P-DQN, and DQN during the test session.

We compare the average normalized test results of our proposed MP-DQN method with P-DQN, DQN, and Nearest SBS+ Random Power in Figure 6a,b and Figure 7 by considering the total time steps with 100 realizations. In Figure 6a, the average standardized test reward has been shown for all methods where maximum average results (around 1.26) for all timesteps are generated by our proposed method MP-DQN. The second height test reward is produced from P-DQN while the nearest SBS with random power for the UE method gives the worst results. In addition, the average normalized energy efficiency for test sessions has been depicted in Figure 6b for all discussed methods. The energy efficiency of our proposed method is approximately 9.89%, 94.7%, and 160.44% better than P-DQN, traditional DQN, and distance-based association methods, respectively during the whole test period. In addition, the average UE throughput by the normalized process has been illustrated in Figure 7 for all methods. According to Figure 7, the average normalized system throughput of our proposed method MP-DQN is approximately 4.27 which is 12.36%, 44.74%, and 19.607% better results compared to P-DQN, DQN, and the nearest distance with random power allocation algorithms. The summary of test results for all methods including the proposed method is presented in Table 4.

### 5.2. Simulation Results Considering UE’s Mobility

**Figure 8:**
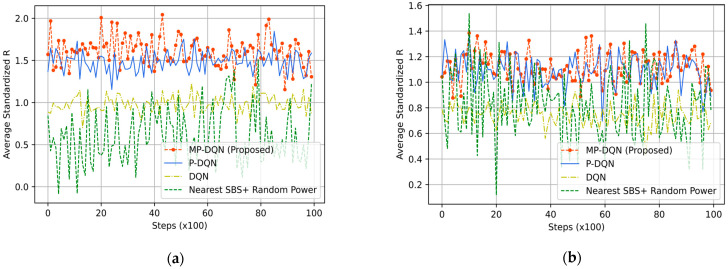
Average standardized reward based on (**a**) RFO and (**b**) RFT from proposed method (MP-DQN), P-DQN, and DQN and Nearest SBS + random power.

**Figure 9:**
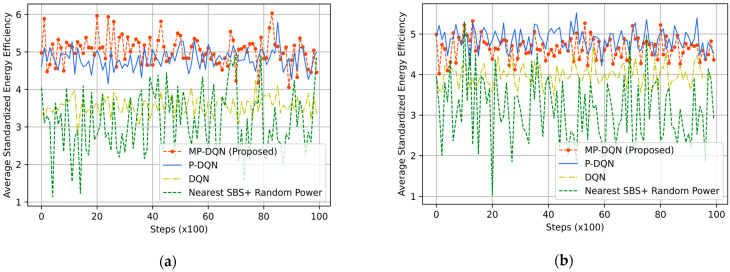
Average standardized energy efficiency based on (**a**) RFO and (**b**) RFT from proposed method (MP-DQN), P-DQN, and DQN and Nearest SBS + random power.

**Figure 10:**
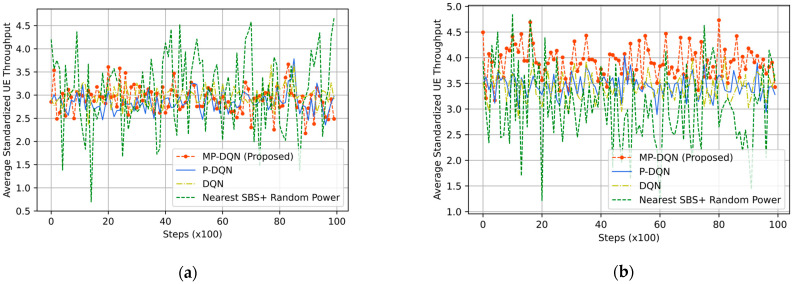
Average standardized UE throughput based on (**a**) RFO and (**b**) RFT from proposed method (MP-DQN), P-DQN, and DQN and Nearest SBS + random power.

**Figure 11:**
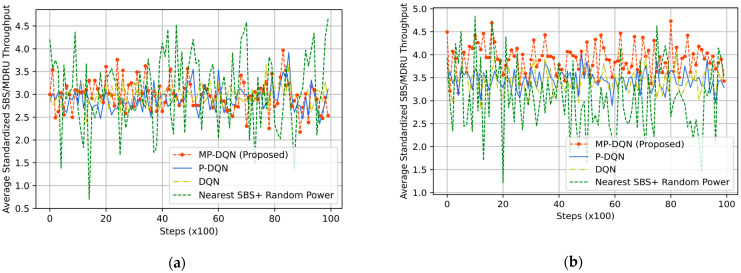
Average standardized SBS throughput based on (**a**) RFO and (**b**) RFT from proposed method (MP-DQN), P-DQN, and DQN and Nearest SBS + random power.

Due to its ability to remember previous activities, the GM mobility model is not stateless. It is appropriate for moveable UEs such as robots, cars, UGV, etc. We have illustrated average standardized reward, emergency efficiency, UE throughput, and SBS/MDRU throughput in Figure 8, Figure 9, Figure 10 and Figure 11, respectively. In addition, simulation results based on RFO and RFT have been illustrated in Figure Xa and Figure Xb, respectively, where X is within 8 to 11. Each UE’s average velocity has been considered 10 km/h with a random direction. In Figure 8a, the average standardized rewards mean is 0.6, 0.98, 1.48, and 1.61 from Nearest SBS + random power, DQN, PDQN, and MPDQN, respectively, based on the RFO. When the simulation is run with the RFT, the MPDQN generates (mean) 1.12, while PDQN and DQN produce 1.08 and 0.74, respectively illustrated in Figure 8b. Average standardized emergency efficiencies for all algorithms have been illustrated in Figure 9a,b according to RFO and RFT, respectively. In Figure 9a, MPDQN gives 5.03 while PDQN, DQN, and Nearest SBS + random power produce 4.81, 3.52, and 3.13, respectively. Furthermore, the MPDQN and PDQN generate almost similar energy efficiency that is better than the DQN and Nearest SBS + random power illustrated in Figure 9b.

For evaluating the IoRT network, the QoS of UE is the crucial parameter that directly depends on the downlink throughput of UE in each time slot. In Figure 10, we have depicted the average standardized UE throughput. When we have utilized the RFO, the means of average standardized UE throughputs are 2.92, 2.85, and 2.97 from MPDQN, PDQN, and DQN, respectively. However, the Nearest SBS + Random power generates 3.08, as shown in Figure 10a. We have illustrated the simulation results using the RFT in Figure 10b. The mean of average standardized UE throughput is 3.05 (similar to Figure 10a) by Nearest SBS + Random power, while DRL-based algorithms generate better results. Hence, the design of an appropriate reward function is the key factor in DRL-based problem formulation. The proposed method (MPDQN) gives 3.91, which is the best UE throughput compared to PDQN (3.44) and DQN (3.40). Another key factor of two-tier HeNet is the backhaul connection from MBS to SBS/MDRU, which depends on the throughput of SBS/MDRU illustrated in Figure 11. The proposed method with the RFT outperforms others that are clearly shown in Figure 11.

**Figure 12:**
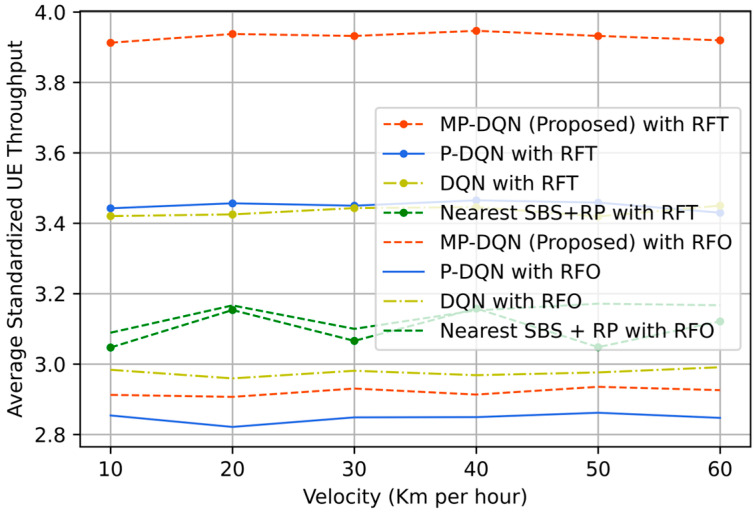
The mean of average standardized UE throughput based with proposed and old reward function from proposed method (MP-DQN), P-DQN, and DQN and Nearest SBS + random power.

In Figure 12, the mean of average standardized UE throughput has been presented concerning the velocity range from 10 Km/h to 60 Km/h. With RFO, the average standardized UE throughput means approximately 3.10, 2.95, 2.92, and 2.84 from Nearest SBS + random power, DQN, MPDQN, and PDQN, respectively for all velocities until 60 Km/hour. In contrast, those are around 3.10, 3.40, 3.44, and 3.93 for Nearest SBS + random power, DQN, PDQN, and MPDQN, respectively, when adopting the RFT. The results are varied for the Nearest SBS + random power method due to the random power allocations. In our simulations, the discrete action (user association) selects the SC, and the continuous action allocates the power from SBS according to user association in every time step. As a result, the increment of velocity does not impact the simulation results. It is shown that the proposed method with the proposed reward function RFT gives a better result compared to others (Nearest SBS + random power, DQN, and PDQN).

The proposed reward function RFT consists of three main factors of a two-tier IoRT network (i) the energy efficiency, (ii) the QoS of UE, and (iii) the QoS of SBS/MDRU, while the original reward function RFO mainly depends on the average standardized energy efficiency and throughput of UE. As a result, DRL algorithms with the proposed reward function produce better results in contrast to DRL algorithms with the original reward function. The proposed method (MP-DQN) performs better than other algorithms due to the solution of excessive parameterization of P-DQN. In summary, MP-DQN with the proposed reward function RFT outperforms PDQN, DQN, and Nearest SBS + random power in reward, average energy efficiency, average system throughput, and average SBS throughput for various velocities of UE.

## 6. Conclusions

For the PDM, authorities deploy various UE such as UGVs, UAVs, UUVs, health care robots, and smartphones via IoRT to collect information in affected areas, where wireless network, especially 4G/LTE/5G and beyond, works as a backbone. Few SBSs of HetNet can be damaged due to the disaster. Hence, the deployment of MDRU to replace malfunctioning SBS is well-established nowadays. In addition, the electric power crisis is a big challenge for PDM. Therefore, power optimization of HetNet by satisfying all UE demands has paid great attention to research. In this article, we have examined UE association and power allocation of SBS/MDRU to optimize the energy efficiency, UE throughput, and SC throughput of the downlink without knowing the environmental priori knowledge while taking into account the backhaul link and QoS guarantee for stationary and movable UE in MDRU aided two-tier HetNet, which are nonconvex, NP-hard, as well as a hybrid action space problem. We have proposed MP-DQN, which is model-free as well as hybrid action space-based DRL algorithm. The simulation results of the proposed method (MP-DQN) have been compared with two DRL-based algorithms (P-DQN and DQN) and the nearest distance-based SBS with random allocation power. During the whole test period considering the stationary UE, our suggested method’s energy efficiency was around 9.89%, 94.7%, and 160.44% better than P-DQN, standard DQN, and distance-based association approaches, respectively. When the problem formulation by considering the modified Gauss–Markov UE mobility model has been investigated, we have proposed a new reward function RFT that is dependent on (i) the average standardized energy efficiency, (ii) the QoS of UE, and (iii) the QoS of SC; however, the original reward function RFO consists of average standardized energy efficiency and throughput of UE. Hence, DRL algorithms with the RFT are superior outcomes to those with the RFO. At various velocities, MP-DQN with the RFT outperforms PDQN, DQN, and Closest SBS + random power regarding reward, average energy efficiency, average system throughput, and average SC throughput.

## Figures and Tables

**Table 1:** Notation summary.

Notation	Definition
M,N,K and U	set of SBSs, MRDUs, SCs and UEs
M, N, K and U	Total number of SBSs, MDRUs, SCs, and UEs
$F_{w}$	Set of subchannels allocated to $u^{th}$ UE
$S_{k}(t)$	The SC serving the $u^{th}$ UE at time slot t
$B_{sub}$	Subchannel bandwidth
$N_{T}$	The number of antennas on MBS
$P_{total}(t)$	Total consumed power by active SCs
$g_{k,u,f}(t)$	The channel gain from $k^{th}$ SC to $u^{th}$ UE with $f^{th}$ subchannel at time slot t
$h_{k,u,f}(t)$	The channel coefficient from $k^{th}$ SC to $u^{th}$ UE in $f^{th}$ subchannel at time slot t
$P_{{SC}_{k},max}$	The maximum power available of $k^{th}$ SC
$p_{k,u,f}(t)$	Emitting power from $k^{th}$ SC to $u^{th}$ UE in $f^{th}$ subchannel at time slot t
$\left|K^{active}(t)\right|$	Total quantity of active SCs at time slot t
$\sigma^{2}$	Noise power
$I_{u,f}(t)$	Interference observed by $u^{th}$ UE in subchannel *f* at time slot t
$C_{k}(t)$	The set of UEs in cluster k at time slot t
$c_{ku}(t)$	Link indicator between $k^{th}$ SC and $u^{th}$ UE at time slot t
$SINR_{uf}(t)$	SINR for $u^{th}$ UE in the $f^{th}$ subchannel at time slot t
$\upsilon_{u}$	Capacity threshold for $u^{th}$ UE
$D_{k}^{SC}$	Maximum downlink data rate for $k^{th}$ SC

**Table 2:** Simulation Parameters.

Parameter	Value
Carrier frequency	2 GHz
Subchannel bandwidth	15 kHz
Number of subchannels	3
Number of subchannels per user	1
MBS antenna array size	100
MBS beamforming group size	20
The radius of the entire network	500 m
Number of SBS	2
Number of MDRU	1
Number of UE	5
SINR threshold of UE	1 for each UE
Rayleigh channel coefficient	h~CN(0,1)
Path loss model from MBS to SBS	$19.77+3.91\times {log}_{10}d_{k}$ in dB and $d_{k}$ in km
Path loss model for SBS to UEs	$30.53+36.71\times {log}_{10}d_{k,u,t}$ in dB and $d_{k,u,t}$ in km at time t
Noise power spectral density	174 dBm/Hz
Maximum transmit power of SBS	24 dBm
Maximum cluster size	3
Transmit power of MBS	43 dBm
Operational power of SBS	0.5 W
Operational power of MBS	130W

**Table 3:** The hyperparameter of MP-DQN.

Parameters	MP-DQN Q Network	MP-DQN Actor	P-DQN Q Network	P-DQN Actor	DQN
Learning rate	10^−4^	10^−5^	10^−5^	10^−5^	10^−3^
Exploration	e-greedy	Ornstein-Uhlenbeck noise	e-greedy	Ornstein Uhlenbeck noise	e-greedy
Number of Outputs	${|A}_{d}|$	$U.{|A}_{d}|$	${|A}_{d}|$	$U.{|A}_{d}|$	${{|A}_{d}|*L}^{U}$
Hidden layer	ReLu, 1024 ReLu, 512 ReLu, 256	ReLu, 1024 ReLu, 512 ReLu, 256	ReLu, 512 ReLu, 128	ReLu, 256	ReLu, 512 ReLu, 128
	Relu 128	Relu 128	Relu 16		Relu 16
Number of Inputs	$U+U.{|A}_{d}|$	U	$U+U.{|A}_{d}|$	U	U

**Table 4:** The average normalized value of implemented methods.

Methods	Average Reward	Average Energy Efficiency	Average UE Throughput
MP-DQN (Proposed)	1.26	6.83	4.27
P-DQN	0.92	6.21	3.80
Nearest SBS+ Random Power	−0.75	2.62	3.57
DQN	0.118	3.51	2.95

## Data Availability

Not applicable.
